# Subxifoid *versus* transthoracic thoracoscopic lobectomy: Results of a retrospective analysis before and after matching analysis

**DOI:** 10.1111/1759-7714.13778

**Published:** 2021-03-10

**Authors:** Claudio Andreetti, Valentina Peritore, Mohsen Ibrahim, Antonio Gagliardi, Giacomo Argento, Giulio Maurizi, Leonardo Teodonio, Nicola Serra, Erino Angelo Rendina, Mario Santini, Alfonso Fiorelli

**Affiliations:** ^1^ Thoracic Surgery Unit, Sant'Andrea Hospital University of Rome La Sapienza Rome Italy; ^2^ Department of Molecular Medicine and Medical Biotechnology University Federico II of Naples Naples Italy; ^3^ Thoracic Surgery Unit University of Campania Luigi Vanvitelli Naples Italy

**Keywords:** Conventional thoracoscopy, lobectomy, lung cancer, subxifoid thoracoscopy

## Abstract

**Background:**

Here, we report our initial experience with subxifoid video‐assisted thoracoscopic surgery (SVATS) lobectomy for the management of primary lung cancer, and compared the outcomes of SVATS with those of conventional transthoracic VATS (CVATS) lobectomies to validate its feasibility and usefulness.

**Methods:**

The clinical data of consecutive patients undergoing VATS lobectomy via SVATS or CVATS for lung cancer were retrospectively compared. The endpoints were to evaluate the statistical differences in surgical results, postoperative pain (measured with visual analog scale [VAS] scores at 8 hours, Day 1, Day 2, Day 3, at discharge, one month and three months after surgery) and paresthesia (measured at one‐ month, and three months after surgery). The two groups were compared before and after matching analysis.

**Results:**

Our study population included 223 patients: 84 in the SVATS and 139 in the CVATS group. The two groups were not comparable for sex (*P* = 0.001), preoperative comorbidity as cardiopathy (*P* = 0.007), BMI value (*P* = 0.003), left‐sided procedure (*P* = 0.04), tumor stage (*P* = 0.04), and tumor size (*P* = 0.002). These differences were overcome by propensity score matching (PSM) analysis that yielded two well‐matched groups which included 61 patients in each group. Surgical outcomes including blood loss, hospital stay and complications were similar before and after matching analysis, but SVATS compared to CVATS was associated with longer operative time before (159 ± 13 vs. 126 ± 6.3, *P* < 0.0001), and after matching analysis (161 ± 23 vs. 119 ± 8.3; *P* < 0.0001) and significant reduction of postoperative pain during the different time‐points (*P* < 0.001), and paresthesia at one (*P* = 0.001), and three months (*P* < 0.0001).

**Conclusions:**

SVATS lobectomy is a feasible and safe strategy with surgical outcomes similar to CVATS lobectomy but with less postoperative pain and paresthesia.

**Key points:**

**Significant findings of the study:**

- Subxifoid thoracoscopic lobectomy is a feasible and safe procedure, with potential benefits in terms of postoperative pain and paresthesia compared to conventional thoracoscopic lobectomy
- Our results showed that surgical outcomes including blood loss, hospital stay, morbidity and mortality are similar but subxifoid thoracoscopy was associated with significant reduction of postoperative pain and paresthesia.

**What this study adds:**

- Subxifoid thoracoscopy is a safe procedure; compared to conventional transthoracic thoracoscopy, it avoids intercostal incisions, and spares nerve trauma, resulting in a reduction of postoperative pain and paresthesia.

## Graphical abstract

Subxifoid thoracoscopy is a safe procedure compared to conventional transthoracic thoracoscopy. It avoids intercostal incision and spares nerve trauma, resulting in a reduction of postoperative pain and paresthesia.

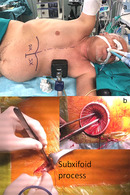

## Introduction

Since its introduction in the early 1990s, video‐assisted thoracoscopic surgery (VATS) has become the preferred approach for the management of early stage lung cancer. Compared to standard thoracotomy, VATS is associated with a significant reduction in postoperative pain, morbidity and mortality, and hospitalization.^1^, ^2^, ^3^, ^4^ Conventional VATS (CVATS) is performed through single or multiple transthoracic incisions that may injure the intercostal nerves, resulting in chest wound pain and paresthesia. More recently, the subxiphoid VATS (SVATS) approach has been proposed as an alternative to CVATS for a variety of thoracic procedures including lung cancer resection.^4^, ^5^, ^6^, ^7^, ^8^, ^9^, ^10^, ^11^ The procedure is performed using only a subxiphoid incision, through which the specimen is also retrieved. Since the intercostal nerve is lacking in the subxiphoid area, this strategy may reduce acute and chronic chest wound pain.^12^, ^13^ However, SVATS is a demanding procedure which is performed in very few high‐volume centers. Thus, there are still concerns regarding the wide feasibility and real advantages of SVATS compared to CVATS.^14^

In this study, we report our initial experience with SVATS lobectomy for the management of lung cancer. We evaluated the validity of this approach by comparison of surgical outcomes, postoperative pain and paresthesia of SVATS lobectomies with those of CVATS lobectomies. This article is presented in accordance with the STROBE reporting checklist which is provided as an additional file.

## Methods

### Study design

This was a retrospective comparative study of patients who underwent lobectomy for management of lung cancer via either the SVTAS or CVATS approaches between January 2015 and January 2020. The patients were not randomized, but underwent SVATS or CVATS at the surgeons' discretion. The end‐points of the study were to evaluate: (i) the feasibility and safety of the SVATS approach by comparison of the surgical outcomes of SVATS with those of CVATS lobectomies; and (ii) whether SVATS was associated with potential benefits over CVATS with regard to postoperative pain and paresthesia.

The study design was approved by the Local Ethics Committees of University of Campania Luigi Vanvitelli (code number: 326‐20); all patients gave their written informed consent for the surgery, and were aware that all their data could be used anonymously for scientific purposes only.

### Participants

The clinical data of all patients undergoing lobectomy for lung cancer via SVATS or CVATS approach and with complete follow‐up regarding surgical outcomes, postoperative pain and paresthesia were included in the study. We excluded: (i) patients undergoing open lobectomy; (ii) patients undergoing VATS resection different from lobectomy (ie, wedge resection, segmentectomy or bilobectomy) or undergoing lobectomy with concomitant decortication and/or chest wall injury or resection; (iii) patients with a history of previous thoracic surgical procedures and/or of chronic pain, or taking regular analgesics; and (iv) patients with incomplete follow‐up.

The choice of approach (CVATS or SVATS) was made by surgeons based on tumor and patient characteristics. Since 2016, with increasing experience in VATS, lobectomy has been performed using a SVATS approach in selected patients. Indications for SVATS were peripheral small tumors, without lymph node involvement. Patients with pleural adhesions, BMI >30 kg/m^2^, and cardiac diseases including cardiomegaly and arrhythmia were not evaluated for SVATS.

All patients received the same preoperative workup including computed tomographic (CT) scanning, integrated with positron emission tomographic (PET‐CT) scanning, and standard cardiopulmonary functional tests. Invasive mediastinal procedures were carried out only in cases of suspicion of mediastinal involvement on radiological examination. For each patient, demographic data, ie, age at operation, gender, body mass index (BMI); preoperative comorbidity; pulmonary function status; tumor characteristics (ie, side, size, stage); operative outcomes (ie, operative time, blood loss, intraoperative complications); postoperative results (ie, output drainage, length of chest drainage, length of hospital stays, morbidity and mortality); and postoperative pain and paresthesia were recorded.

### Surgical procedure

CVATS and SVATS were performed under general anesthesia, and single‐lung ventilation. Surgical equipment included endoscopic articulating linear cutters, long curved endoscopic surgical instruments, curved suction, and a 10 mm 30 grade thoracoscope. Energy‐based devices and surgical endoclips were also used to divide small vessels and/or thin portions of the fissures, and for lymphadenectomy.

CVATS lobectomy was performed exclusively via a standard three‐port approach. The patient was positioned in lateral decubitus and the surgeon was positioned in front of the patient. A 4 cm incision was carried out within the fourth intercostal space on the anterior axillary line, and protected by a wound retractor. This access was used for working instruments, staplers, and for retrieving specimens. A 10 mm incision for the camera was performed within the seventh intercostal space in the mid‐axillary line, and a 20 mm incision for grasping the lung, dissection and for stapling was made within the sixth or seventh intercostal space in the posterior axillary line.

**Figure 1:**
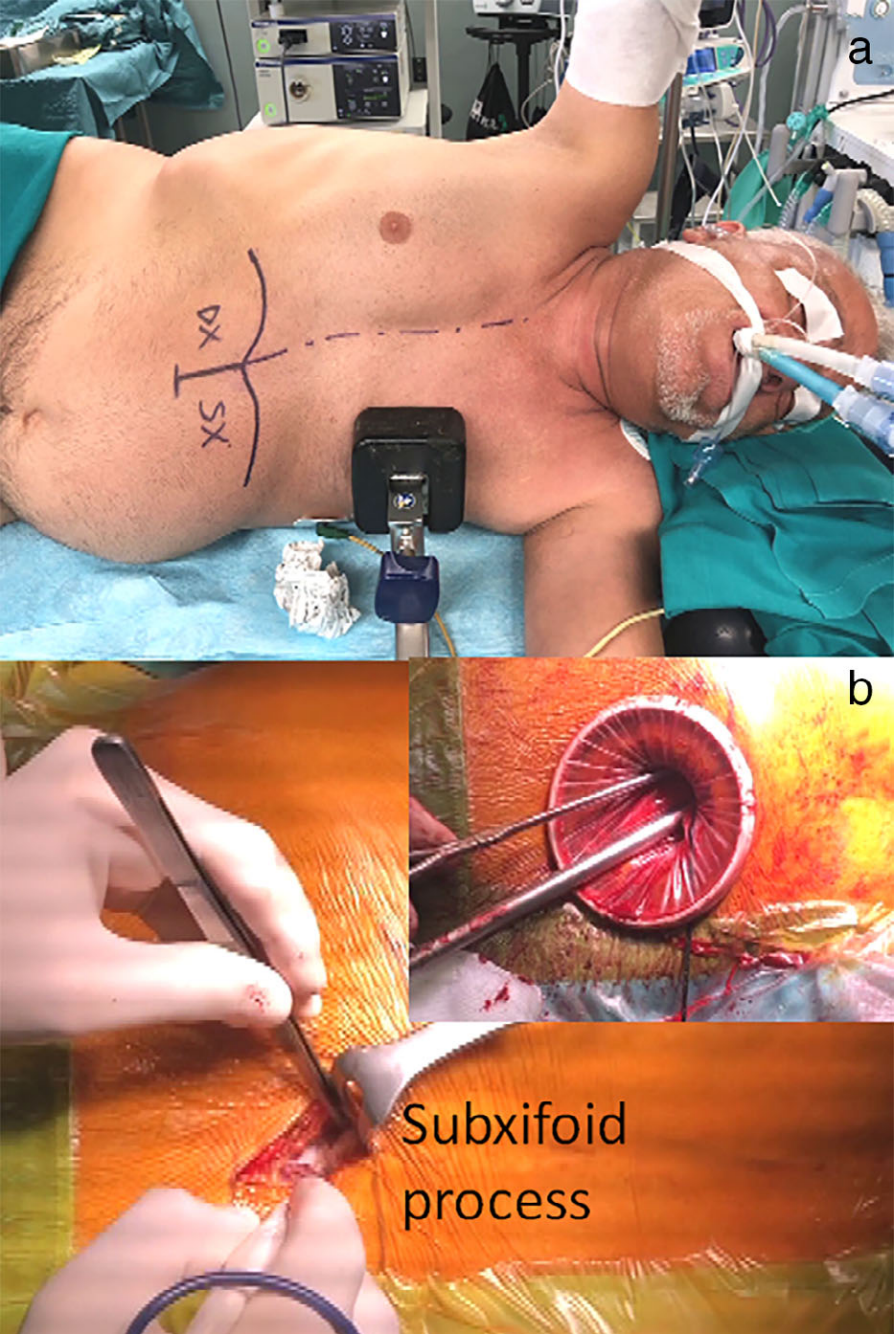
(**a**) Patient position; and (**b**) surgical incision for subxifoid thoracoscopy.

SVATS was performed using a single incision in the subxiphoid space. The patient was placed in a lateral decubitus position with a 30 degree lateral inclination (Fig 1a). Both the surgeon and the assistant were placed contralaterally to the surgical lung. A 4 cm long transversal incision was made below the xifoid process. The skin, subcutaneous tissue and fascia of the rectus abdominis muscle were cut to expose the xiphoid cartilage. The tunnel close to the xifoid process was bluntly dissected with a finger, and the pleural cavity was entered above the diaphragm. A wound protector was then inserted to obtain an optimal view (Fig 1b).

**Figure 2:**
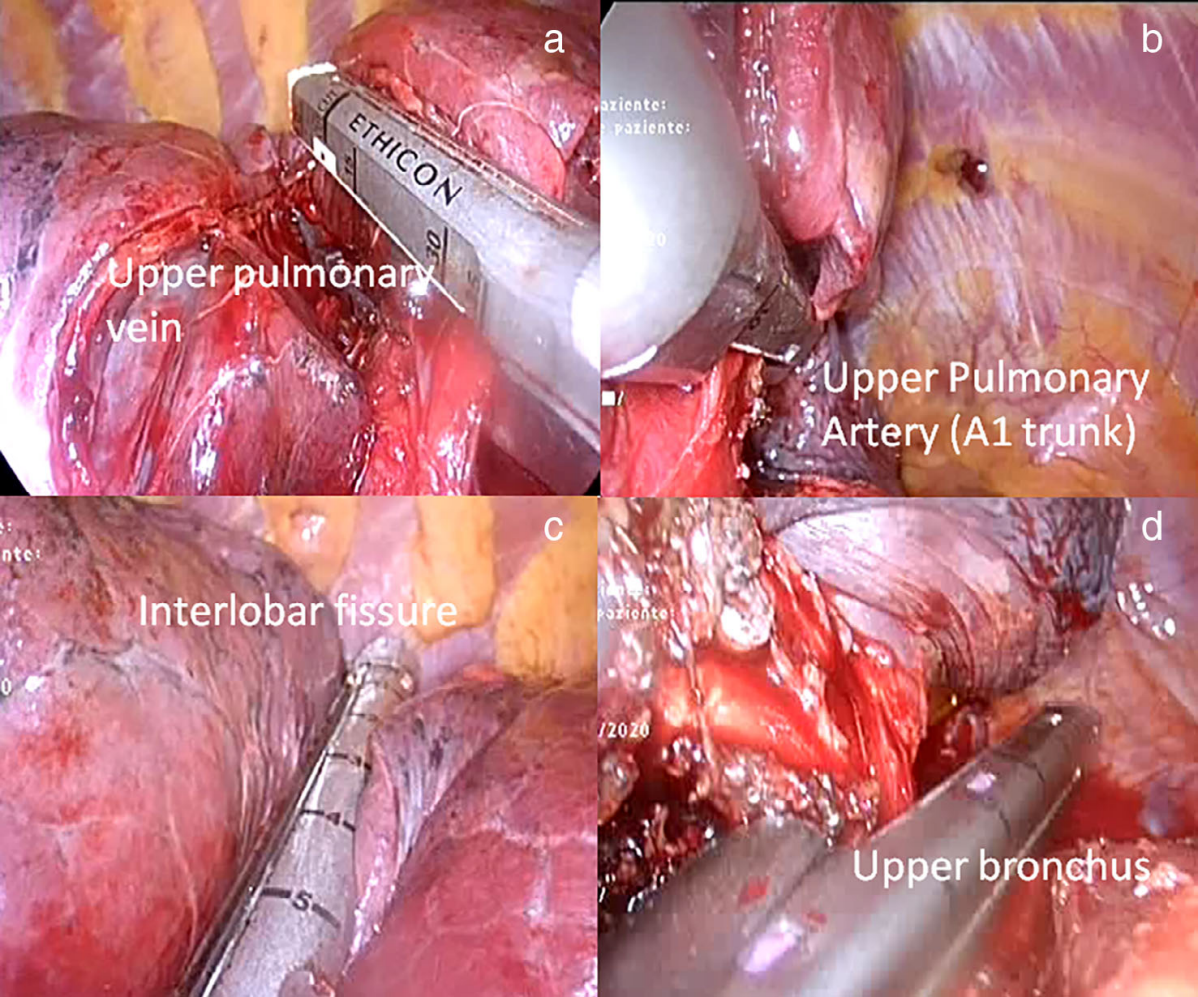
Main steps for subxifoid thoracoscopy upper right lobectomy: sequential resection with stapler of target (**a**) pulmonary veins; (**b**) arteries; (**c**) fissures; and (**d**) bronchus.

Both procedures (CVATS and SVATS) followed the classic principles of pulmonary resections. The first step was to explore the pleural cavity for any unexpected disease and to resect adhesions. Next, the lobectomy was performed. The target pulmonary veins (Fig 2a), arteries (Fig 2b), fissures (Fig 2c), and bronchus (Fig 2d) were divided sequentially, with appropriate endoscopic staplers. Then, the specimen was removed using an endoscopic retrieval bag, and extended lymphadenectomy performed to include stations 2R, 4R, 7, 8, and 9 for right‐sided cancers and stations 4L, 5, 6, 7, 8, and 9 for left‐sided cancers. The bronchial stump was checked under water for air leaks, and a drain was inserted through the camera port for CVATS or via subxiphoid incision for SVATS and positioned to the thoracic apex. It was then connected to a drainage bottle.

After surgery, the patient was usually transferred to an intensive care unit and the next day to the postoperative ward. The drain was removed when there was no air leakage, and the daily output was less than 300 mL.

### Surgical outcome

Surgical outcome was assessed by collecting operative and postoperative data. Operative data included operative time (minutes), intraoperative blood loss (mL), and complications (ie, vascular injury, cardiac complications, etc). Postoperative data included output drainage, length of chest drainage, length of hospital stay and morbidity and mortality.

### Postoperative pain control

The protocol for postoperative pain management was the same for each group. Pre‐emptive wound analgesia with ropivacaine was performed before incisions. At the end of the operation, a multilevel intercostal nerve block using ropivacaine (7.5 mg diluted in 20 mL of saline), 4 mL for each intercostal space, was performed including the intercostal level of the incision and one level above and below. During the postoperative course, oral paracetamol (1 g), and oral Ketorolac (20 mg) was administered every 6 hours. If the visual analog scale (VAS) score was higher than 4, intravenous injection of morphine (0.1 mg/kg) was administered.

### Postoperative pain evaluation

Maximum pain scores were evaluated using the VAS with 11‐point levels, ranging from 0 (no pain) to 10 (the worst pain experienced). Pain was evaluated postoperatively at eight hours, on days 1, 2, and 3 and at discharge. All patients were subsequently followed‐up in the outpatient department by general enquiry at the time of the first and third months to evaluate any residual pain.

### Postoperative paresthesia evaluation

Paresthesia was assessed using data from previously reported studies.^15^, ^16^ The most common characteristics were “pins and needles”, a sensation of “abnormal swelling”, and “numbness”. They were graded as follows: mild, moderate, and severe. The paresthesia was recorded one and three months after surgery.

### Study size

We calculated our sample size based on the primary outcome measure as the VAS score. Based on previous studies,^17^, ^18^ a sample size of 40 patients per each group was calculated, assuming an intention to detect an effect size of 0.65 in the mean difference of the VAS score with 80% power and a type I error rate of 0.05. In anticipation of excluding patients after propensity score matching (PSM) analysis, we extended the number of patients before any comparative analysis.

### Statistical analysis

The Kolmogorov‐Smirnov test and graphic histograms were used to check the normality/skewness of continuous variables data in subgroups before further analysis, and appropriate statistical tests were chosen accordingly. Data are summarized as mean and standard deviation (SD) for continuous variables, and absolute number and percentage for categorical variables. We used a Student's *t*‐test for normally distributed variables to compare means, the Mann‐Whitney test to compare non‐normally distributed variables, and a chi‐square test to compare categorical data. Repeated measures analysis of variance (ANOVA) test, corrected with Bonferroni post‐hoc test, if indicated, was used for comparison of symmetric continuous variables measured at different time‐points.

To minimize the influence of clinical confounders on outcomes, PSM analysis between the two groups was performed to create comparable groups of patients. A 1–1 ratio was used and the propensity score was constructed by using the following covariates: age (<70 or ≥70 year‐old); BMI (< 25 or ≥25); ppoFEV1% (≤40% or >40%); cardiac disease (yes or no); involved side and type of resection (right upper or right lower lobectomy, left upper lobectomy and left lower lobectomy); tumor size (≤30 mm or >30 mm); stage (stage I–II or stage III–IV); and major postoperative complications (yes or no). Specifically, we sought to match each SVATS patient to CVATS patient who had a propensity score that was identical to nine digits. If this match could not be found, the algorithm then proceeded sequentially to the next highest digit match on propensity score to make “next best” matches, in a hierarchical sequence until no more matches could be made. Once a match was made, previous matches were not reconsidered before making the next match. More detailed information is reported as a supplementary file. A *P*‐value < 0.05 was considered statistically significant. MedCalc statistical software (Version 12.3, Mariakerke, Belgium) and PASS 11 (NCSS, LLC, Kaysville, Utah, USA) were used for the analysis.

## Results

**Figure 3:**
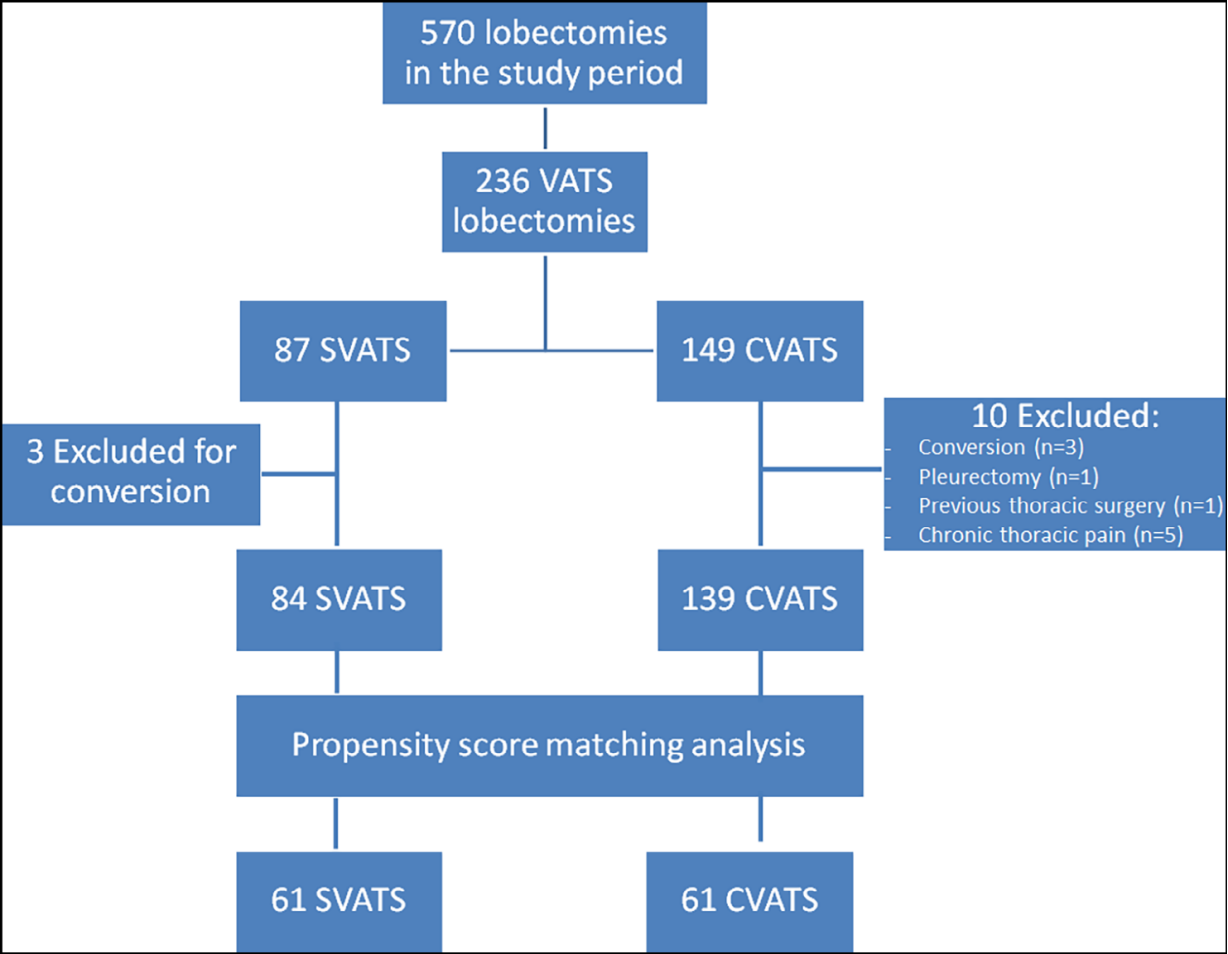
Flow chart of the study.

In the study period, 570 patients underwent lobectomy for cancer, and 236 of these were performed via CVATS or SVATS. In the CVATS group, 10 patients were excluded. In one case lobectomy was associated with the resection of parietal pleura, while in three there was a conversion to thoracotomy due to a vascular (*n* = 2) or bronchial lesion (*n* = 1). Five patients presented with chronic postoperative pain, and one had previous chest surgery. In the SVATS group, three patients were excluded as they were converted to thoracotomy for vessel injury (*n* = 1), for technical difficulties and bleeding (*n* = 1), and for arrhythmia and hypotension (*n* = 1). Thus, our study population included a total of 223 patients for final analysis: 84 in the SVATS group, and 139 in the CVATS group. The flow chart of the study is summarized in Fig 3.

The CVATS compared to the SVATS group had a higher percentage of male patients (71% vs. 51%; *P* = 0.001); patients with cardiopathy (15% vs. 3%; *P* = 0.007); and with a higher BMI (28 ± 4.9 vs. 22 ± 3.8; *P* = 0.003) (Table 1). Yet, CVATS had a higher rate of patients who underwent right (30% vs. 9%; *P* = 0.0003), and left lower lobectomies (17% vs. 8%; *P* = 0.04); patients with larger tumors (37 ± 11 vs. 23 ± 0.8; *P* = 0.002) and those at an advanced stage (9% vs. 3%; *P* = 0.04) (Table 2). The propensity score yielded two well‐matched groups including 61 patients in each group. The matched groups were comparable with respect to age, sex, BMI, preoperative, and tumor characteristics (Table 1). Tumor histology, stage, number of retrieved lymph nodes, and of resected nodal stations and nodal status were also comparable between both groups (Table 2). A R0 resection was achieved in all patients.

**Table 1:** Patient characteristics before and after matching analysis

Variable	All	SVATS	CVATS	*P*‐value
Before matching analysis				
Number of patients	223	84	139	—
Age (year‐old)	63.3 ± 7.6	63.3 ± 1.8	62.9 ± 7.2	0.82
Sex (male)	142	43 (51%)	99 (71%)	0.001
BMI	26 ± 5.9	22 ± 3.8	28 ± 4.9	0.003
Preoperative comorbidity COPD Cardiopathy Diabetes Hypertension	71 24 11 16	26 (31%) 3 (3%) 5 (6%) 6 (7%)	45 (32%) 21 (15%) 9 (6%) 15 (11%)	0.74 0.007 0.87 0.36
Functional data FEV1 6MWT	2.3 ± 0.9 383 ± 59	2.3 ± 0.4 389 ± 60	2.2 ± 0.6 376 ± 56	0.73 0.15
After matching analysis				
Number of patients	122	61	61	—
Age (year‐old)	63.3 ± 4.6	63.1 ± 3.8	63.2 ± 8.2	0.92
Sex (male)	61 (50%)	30 (49%)	31 (51%)	0.85
BMI	22 ± 6.8	22 ± 3.8	22 ± 8.9	0.84
Preoperative comorbidity COPD Cardiopathy Diabetes Hypertension	37 (30%) 5 (4%) 11 (9%) 10 (8%)	18 (29%) 2 (3%) 4 (6%) 4 (6%)	19 (31%) 3 (5%) 7 (11%) 6 (10%)	0.79 0.63 0.33 0.49
Functional data FEV1 6MWT	2.3 ± 0.6 384 ± 67	2.3 ± 0.5 385 ± 41	2.2 ± 0.8 383 ± 75	0.85 0.64

**Table 2:** Pathological data of study population

Variable	All	SVATS	CVATS	*P*‐value
Before matching analysis				
Number of patients	223	84 (38%)	139 (62%)	—
Type of lobectomy RUL ML RLL LUL LLL	85 (38%) 21 (9%) 50 (22%) 38 (17%) 29 (14%)	50 (59%) 10 (12%) 8 (9%) 10 (12%) 6 (8%)	35 (25%) 11 (8%) 42 (30%) 28 (20%) 23 (17%)	<0.0001 0.30 0.0003 0.11 0.04
Histology Squamous carcinoma Adenocarcinoma Large cell carcinoma	71 (32%) 128 (57%) 24 (11%)	27 (32%) 50 (59%) 7 (9%)	44 (32%) 78 (56%) 17 (12%)	0.93 0.61 0.36
Tumor size (mm)	35 ± 13	23 ± 0.8	37 ± 11	0.002
Pathological status Stage I Stage II Stage III	180 (81%) 27 (12%) 15 (7%)	73 (86%) 9 (11%) 2 (3%)	108 (77%) 18 (13%) 13 (9%)	0.11 0.62 0.04
Nodal station	6.3 ± 0.7	6.3 ± 0.6	6.4 ± 0.9	0.85
Nodal number	9.3 ± 0.8	9.2 ± 0.7	9.3 ± 1.3	0.75
Nodal upstaging	28	7 (8%)	18 (13%)	0.29
After matching analysis				
Number of patients	122	61	61	
Type of lobectomy RUL ML RLL LUL LLL	67 (55%) 12 (10%) 13 (10%) 16 (13%) 14 (12%)	36 (57%) 7 (12%) 6 (9%) 7 (12%) 5 (8%)	31 (51%) 5 (8%) 7 (11%) 9 (15%) 9 (15%)	0.46 0.54 0.77 0.59 0.25
Histology Squamous carcinoma Adenocarcinoma Large cell carcinoma	40 (33%) 67 (55%) 15 (12%)	19 (31%) 35 (57%) 7 (12%)	21 (34%) 32 (52%) 8 (14%)	0.70 0.58 0.78
Tumor size (mm)	27 ± 0.7	26 ± 0.5	28 ± 0.9	0.25
Pathological status Stage I Stage II Stage III	102 (84%) 15 (12%) 5 (4%)	52 (85%) 7 (12%) 2 (3%)	50 (82%) 8 (13%) 3 (5%)	0.62 0.78 0.64
Nodal station	6.4 ± 0.9	6.4 ± 0.3	6.4 ± 0.3	0.87
Nodal number	9.5 ± 1.4	9.5 ± 1.7	9.5 ± 2.3	0.79
Nodal upstaging	9 (7%)	4 (6%)	5 (8%)	0.73

### Surgical outcomes

The data are summarized in Table 3. There were no significant differences between the SVATS group and the CVATS group in blood loss, drainage time and output, length of hospital stay before and after matching analysis. Operative time was longer in the SVATS compared with the CVATS group in unmatched (159 ± 13 vs. 126 ± 6.3, *P* < 0.0001), and matched populations (161 ± 23 vs. 119 ± 8.3; *P* < 0.0001). The incidence of overall postoperative complications was 4% and 6% in unmatched and matched populations, respectively. The SVATS group compared to the CVATS group showed no statistical differences before (*P* = 0.99) and after matching analysis (*P* = 0.69). There were no perioperative deaths, with 30‐ and 90‐day survival of 100% in both groups.

**Table 3:** Operative and postoperative data

Variables	All patients	SVATS	CVATS	*P*‐value
Before matching analysis				
Number of patients	223	84	139	—
Operation time (hours)	137 ± 29	159 ± 13	126 ± 6.3	<0.0001
Intraoperative blood loss (mL)	208 ± 16	210 ± 13	205 ± 23	0.36
Daily chest drainage volume (mL)	193 ± 49	180 ± 43	200 ± 59	0.31
Chest tube removal (days)	3.4 ± 0.5	3.1 ± 0.7	3.6 ± 0.9	0.31
Length of hospital stay (days)	4.7 ± 1.3	4.5 ± 1.1	4.8 ± 1.0	0.46
Complications (total) Prolonged air leak (>5 days) Atelectasis Atrial fibrillation	8 (4%) 3 (1%) 2 (0.7%) 3 (1%)	3 (3%) 1 (1%) 0 () 2 (2%)	5 (3%) 2 (1%) 2 (1%) 1 (0.7%)	0.99
After matching analysis				
Number of patients	122	61	61	—
Operation time (hours)	137 ± 29	161 ± 23	119 ± 8.3	<0.0001
Intraoperative blood loss (mL)	210 ± 34	208 ± 53	210 ± 29	0.21
Daily chest drainage volume (mL)	190 ± 39	189 ± 34	190 ± 65	0.28
Chest tube removal (days)	3.5 ± 0.6	3.4 ± 0.8	3.5 ± 0.7	0.41
Length of hospital stay (days)	5.2 ± 1.3	5.1 ± 1.4	5.2 ± 1.2	0.31
Complications (total) Prolonged air leak (>5 days) Atelectasis Atrial fibrillation	7 (6%) 3 (2%) 1 (1%) 3 (2%)	3 (5%) 1 (2%) 0 2 (3%)	4 (6%) 2 (3%) 1 (2%) 1 (2%)	0.69

### Postoperative pain

**Figure 4:**
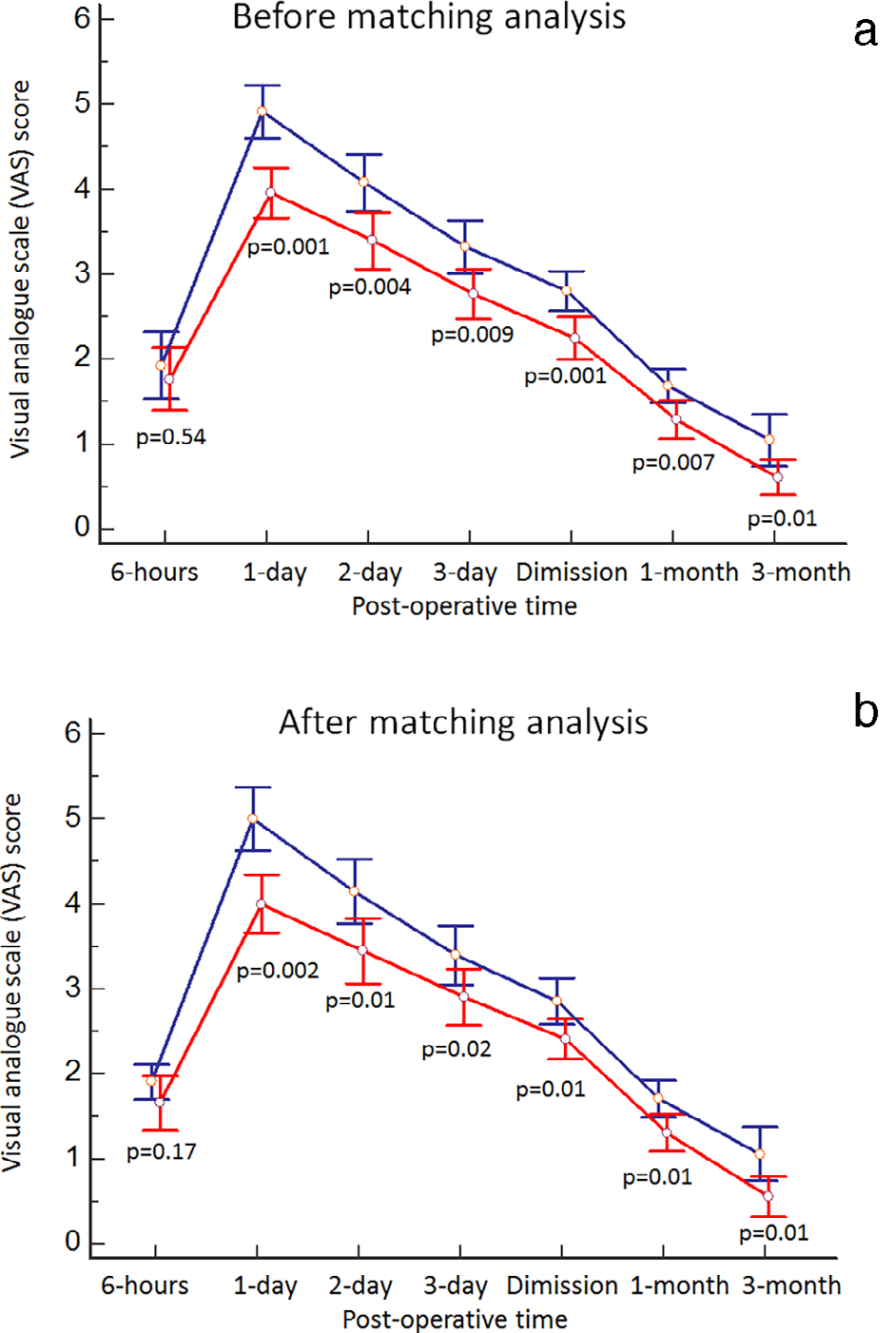
Subxifoid thoracoscopy compared to conventional thoracoscopy was associated with significant reduction of postoperative pain during the entire postoperative follow‐up (*P* < 0.001) (**a**) before 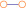, CVATS; 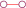, SVATS and (**b**) after matching analysis 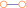, CVATS; 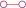, SVATS.

The data are summarized in Table 4. There was no significant difference between SVATS and CVATS in the first eight hours postoperatively (1.7 ± 0.8 vs. 1.9 ± 0.9; *P* = 0.54), while significant differences were found at day 1 (3.9 ± 0.7 vs. 4.9 ± 0.7; *P* = 0.001), day 2 (3.4 ± 0.8 vs. 4.0 ± 0.8; *P* = 0.004), day 3 (2.7 ± 0.7 vs. 3.3 ± 0.8; *P* = 0.009), at discharge (2.2 ± 0.5 vs. 2.8 ± 0.5; *P* = 0.001), one month (1.2 ± 0.5 vs. 1.6 ± 0.4; *P* = 0.007) and three months (0.6 ± 0.5 vs. 1.0 ± 0.7; *P* = 0.01) after surgery (Fig 4a). ANOVA test showed that SVATS was associated with significant reduction of VAS during the entire postoperative follow‐up (*P* < 0.001). Similar results were observed in the matched analysis population (Fig 4b). Additionally, the CVATS group compared to the SVATS group had a higher rate of patients (4% vs. 15%, *P* = 0.04) who needed morphine to manage their pain at a comfortable level (VAS ≤4).

**Table 4:** Postoperative VAS score

VAS scores	SVATS	CVATS	*P*‐value*
Before matching analysis			
8 hours after operation	1.7 ± 0.8	1.9 ± 0.9	0.54
Day 1 after operation	3.9 ± 0.7	4.9 ± 0.7	0.001
Day 2 after operation	3.4 ± 0.8	4.0 ± 0.8	0.004
Day 3 after operation	2.7 ± 0.7	3.3 ± 0.8	0.009
At discharge	2.2 ± 0.5	2.8 ± 0.5	0.001
1 month after operation	1.2 ± 0.5	1.6 ± 0.4	0.007
3 months after operation	0.6 ± 0.5	1.0 ± 0.7	0.01
After matching analysis			
8 hours after operation	1.6 ± 0.6	1.9 ± 0.4	0.17
Day 1 after operation	4.0 ± 0.7	5.0 ± 0.7	0.002
Day 2 after operation	3.4 ± 0.8	4.1 ± 0.8	0.01
Day 3 after operation	2.9 ± 0.7	3.4 ± 0.7	0.02
At discharge	2.4 ± 0.5	2.8 ± 0.5	0.01
1 month after operation	1.3 ± 0.4	1.7 ± 0.3	0.01
3 months after operation	0.5 ± 0.3	1.0 ± 0.6	0.01

* ANOVA test showed that SVATS was associated with significant reduction of VAS during the entire postoperative follow‐up (*P* < 0.001) before and after matching analysis.

### Postoperative paresthesia

Before matching analysis, the incidence of paresthesia in the SVATS compared to the CVATS groups was 20% vs. 42% (*P* = 0.001) one month after surgery, and 6% vs. 34% (*P* < 0.0001) three months after surgery. After matching analysis, paresthesia was also significantly lower in the SVATS than in the CVATS groups one month (19% vs. 41%; *P* = 0.001) and three months (5% vs. 33%; *P* < 0.0001) after surgery. In all cases, the paresthesia level did not limit the patients' daily activities.

## Discussion

Postoperative pain and chest wall paresthesia remain unsolved issues for thoracic surgeons. They also still occur when surgical resection is performed using CVATS. The reduction in the number of ports does not affect postoperative pain, confirming that port location could be more important than numbers.^19^, ^20^, ^21^ Therefore, in the last few years there has been a growing interest in performing VATS using the subxiphoid route in order to avoid intercostal incisions, and spare nerve trauma. Initially, SVATS was used for the management of pneumothorax,^22^, ^23^ and mediastinal tumors.^24^, ^25^ Then, a few high‐volume centers also performed anatomical lung resections using SVATS with satisfactory short‐term results.^4^, ^5^, ^6^, ^7^, ^8^, ^9^, ^10^, ^11^ However, it is still debatable whether SVATS lobectomy presents real advantages over CVATS lobectomy. Four reports, ^5^, ^6^, ^7^, ^8^ summarized in Table 5, previously evaluated this issue with contrasting results as SVATS was associated with shorter hospitalization in two studies,^6^, ^8^ and patients with lower postoperative pain were reported in three.^5^, ^7^, ^8^ The small cohorts of patients, subjectivity of pain reporting, and lack of standardized pain control protocols used were the main limitations of these studies. A Randomized Controlled Trial (RCT), registered in ClinicalTrials.gov. database (NCT03331588) from Shanghai Pulmonary Hospital, Shanghai, China is planning to compare postoperative pain, and quality of life between SVATS and CVATS for lung cancer management.^26^ Patient recruitment was completed last February 2020, but the results are not yet published. Thus, to evaluate the feasibility and outcome of SVATS lobectomy, we retrospectively compared the results of SVATS with those of CVATS.

**Table 5:** Review of papers comparing SVATS versus CVATS lobectomies for lung cancer management

	Surgical outcomes							
Authors	Operative time	LN resection	LHOS	Conversion	Morbidity	Mortality	Postoperative pain	Quality of life
Song *et al*. 2016^5^	Longer for SVATS	None	None	N/A	None	None	Lower for SVATS	N/A
Pfeuty & Lenot 2019^6^	None	None	Lower for SVATS	None	None	None	None	N/A
Yang *et al*. 2019^7^	Longer for SVATS	None	None	None	None	None	Lower for SVATS	N/A
Chen *et al*. 2020^8^	None	None	Lower for SVATS	None	None	None	Lower for SVATS	Better for SVATS
Present series	Longer for SVATS	None	None	None	None	None	Lower for SVATS	N/A

First, our surgical outcomes related to SVATS which included operative time, length of hospital stay (LHOS), postoperative morbidity and mortality were similar to those reported by other authors (Table 6). No cases of conversion were observed, while the incidence of conversion in other series^5^, ^6^, ^7^, ^8^, ^9^, ^10^, ^11^ ranged from 2% to 12%. A possible explanation may be the different patient selection. Our study included highly selected patients who could be considered as “ideal candidates” for SVATS. In fact, they had peripheral and small cancer, without lymph node involvement and pleural adhesions, and low BMIs. Most of the procedures were right‐sided, with no complex resections such as sleeve lobectomy with bronchial or artery resection and reconstruction, and chest wall resection was performed as in other studies.^6^ When we compared the surgical outcomes such as LHOS, postoperative morbidity and mortality between SVATS and CVATS, no significant differences were found, supporting the safety of the SVATS procedure. Our results were in line with those of Song *et al*. ^5^ and Yang *et al*. ^7^ while Pfeuty and Lenot^6^ and Chen *et al*.^8^ found that SVATS was associated with lower LHOS compared to CVATS. The increased use of digital drainage devices and the ERAS program for SVATS in the series by Pfeuty and Lenot,^6^ and the higher rate of wound infection for CVATS group in the series by Chen *et al*.^8^ could explain the different results.

**Table 6:** Review of literature regarding surgical outcomes after SVATS resections

Authors	Resections	Operative time (minutes)	LHOS	Conversion	Morbidity	Mortality
Hernandez‐Arenas *et al*. 2016^9^	Lob: 105 Segm: 48	166.9 ± 12.6	4.3 ± 0.4	13 (8,4%)	Arrhythmia: 20 (13%)	0
Liu *et al*. 2016^10^	Lob: 21 Segm: 5 Wedge: 9 ‐Mediastinal surgery: 5	180	N/A	5 (12,5%)	Total: 4 (10%) Arrhythmia: 2 (5%) Chylothorax: 1 (2.5%) Bleeding: 1 (2.5%)	0
Song *et al*. 2016^5^	Lob: 105	164.97 ± 39.10	5.39 ± 1.31	2 (2%)	Total: 11 (10.5%) PAL: 2 (2%) Chylothorax: 1 (1%) Arrhythmia: 3 (3%) Wound problems: 2 (2%) Bleeding: 1 (1%) Atelectasis: 1 (1%) Pulmonary embolism:1 (1%)	0
Ali *et al*. 2018^11^	Segm: 242 (29 bilateral)	2.14 ± 0.78	4.67 ± 9.54	15 (2.5%)	Total: 21 (8.26%) Bleeding: 1 (0,4%) Change to lob: 3 (1.2%) PAL: 6 (2.4%) Thoracic hematoma: 2 (0.8%) Arrhythmia: 9 (3.7%)	0
Pfeuty & Lenot 2019^6^	Lob: 51 Segm: 20 Pneum: 4	157 ± 37	2 (1–4)	7 (9%)	Total: 18 (9%) Minor: 13 (17%) Major: 5 (7%)	1 (13%)
Yang *et al*. 2019^7^	Lob: 34 Lob + wedge: 3	262.15 ± 40.68	9.23 ± 2.94	1 (2.7%)	Total: 3 (8.11%) Pneumonia: 1 (2.70%) Arrhythmias: 1 (2.70%) PAL: 1 (2.70%)	0
Chen *et al*. 2020^8^	Lob: 459	129.7 ± 2.2	4.24 ± 0.08	9 (1.96%)	Total: 29 (6.3%) Wound infection: 2 (0.44%) Subcutaneous emphysema: 2 (0.44%) Respiratory failure: 2 (0.44%) PAL: 6 (1.31%) Pulmonary embolism: 1 (0.22%) Arrhythmia: 16 (3.49)	0
Present series	Lob: 84	159 ± 13	4.5 ± 1.1	None	Total: 3 (3%) PAL: 1 (1%) Arrhythmia: 2 (2%)	0

Lob, lobectomy; PAL, persistent air‐leaks; Pneum, Pneumonectomy; Segm, segmentectomy; wedge, wedge resection.

Second, SVATS compared to CVATS was associated with a significant reduction in acute postoperative pain during the entire postoperative course. Although a trend was seen during the first eight postoperative hours, the difference become more evident in the following days, and at discharge. In theory, the residual effects of general anesthesia still influenced the pain perception in the immediate postoperative period. Our results were in line with all previous authors but Pfeuty and Lenot^6^ who found no significant difference between SVATS and CVATS in terms of postoperative pain, probably due to the different protocol of locoregional pain management used for the two groups. CVATS patients had a paravertebral catheter with continuous analgesia, during a median of two days, whereas the SVATS patients received a local one‐shot subxiphoid and intercostal block. However, the same authors^6^ found that SVATS was associated with a significant reduction of home morphine use at day seven to manage their pain at a comfortable level (NRS < 3). Similarly, also in our series, CVATS had a higher rate of patients who needed morphine to manage their pain at a comfortable level (VAS ≤4), supporting the evidence of the superiority of SVATS over CVATS for postoperative pain control. Suda *et al*.^24^ also found that the consumption of morphine was significantly reduced after thymectomy performed with SVATS than with CVATS. The damage to the intercostal nerve is the main cause of postoperative pain after thoracic procedures. Using the subxiphoid route does not injure the intercostal nerve during the procedure because there is no bone structure or intercostal nerve in the subxiphoid incision. Yet, a chest drain is not inserted through the intercostal space so it cannot damage the intercostal nerve during patient mobilization. Thus, all these reasons may probably explain the decrease in postoperative pain associated with SVATS. Furthermore, patients had less postoperative pain and lower rates of paresthesia one and three months after SVATS. In theory, SVATS might solve the postoperative chronic persistent pain caused by the intercostal incision experienced by some patients, but more studies are still required to confirm this hypothesis.

Third, from a technical point of view, SVATS remains a more challenging procedure compared to CVATS as shown by the longer operative time observed in our study and other series.^5^, ^7^ Challenges related to SVATS include the long distance between the subcutaneous incision and the hilus of the lung, small operative angle, and interference of surgical instruments. Thus, a BMI >30 kg/m^2^ remains the main contraindication for SVATS^27^ as the presence of subcutaneous and mediastinal fat may impede tunnel creation between the subxiphoid wound and pleural space. Yet, during the left procedure, the intraoperative heart compression caused by the passage of instruments through the subxifoid incision into the left chest wall may induce cardiac pulsation and arrhythmia. To overcome these limits, Pfeuty and Lenot^6^ proposed a new multiportal SVATS using three ports on the left side due to partial cardiac obstruction and relatively difficult access to the subcarinal area, and two ports for right‐sided resections. Based on the authors' experience,^6^ this strategy facilitated the maneuverability of the endostaplers, magnified the endoscopic view, and limited the conflict between camera and other instruments. Thus, the different SVATS approach could explain the lack of differences regarding the operative time between SVATS and CVATS, or the possibility of performing more complex procedures such as sleeve lobectomies.

The present study had several limitations; thus, our results should be evaluated with caution before drawing any definitive conclusions. The small sample size, and selection bias due to the retrospective nature of the study were the main limitations, despite PSM being performed to reduce the intergroup differences. Yet, we compared a single technique incision using SVATS versus a multiport technique incision using CVATS. In theory, the number of incisions rather than their location could affect the results. However, several reports^19^, ^20^, ^21^ have previously compared postoperative pain between single‐ versus multiport VATS and found no significant difference between the two procedures. Finally, SVATS was performed by the same surgeon (CA) while CVATS was performed by different surgeons (CA, MI, GM, and AF); thus, different skills and experiences could affect the results.

In conclusion, this study confirmed that SVATS lobectomy is a feasible and safe procedure with surgical outcomes similar to those of CVATS lobectomy, but with significant advantages in controlling postoperative pain and paresthesia. However, SVATS remains a complex procedure that needs particular skills, and appropriate endoscopic instruments. Thus, it should be performed in a high volume center and by surgeons with considerable experience using the VATS procedure. Furthermore, patient selection is crucial for the success of the procedure. Obviously, our results should be corroborated by further and larger studies to establish the potential and promising outcomes of this strategy.

## Disclosure

All authors disclose no conflict of interest and no funding for this study.

## Supporting information


**Appendix S1:** Supporting Information
