# Supplementary material for: Subxifoid versus transthoracic thoracoscopic lobectomy: Results of a retrospective analysis before and after matching analysis
Source: Thorac Cancer. 2021 Mar 10;12(9):1279–90. doi: 10.1111/1759-7714.13778 (PMC8088929; doi:10.1111/1759-7714.13778)
Supplement: Supplementary file 1 — Appendix S1: Supporting Information [file TCA-12-1279-s001.docx]

**Propensity score matching**

To minimize the influence of clinical confounders on outcomes, propensity score matching between the two groups was performed to create comparable groups of patients. A 1-1 ratio was used and the propensity score was constructed by using the following covariates: age (<70 or ≥ 70 year-old); BMI (< 25 or ≥ 25); ppoFEV1 % (≤40% or >40%); cardiac disease (yes or no); involved side and type of resection (right upper/lower, left upper, left lower) based on the difficult of operation (generally, for SVATS right operation is easier than left, and among left lobectomies upper is easier than lower); tumor size (≤30 mm or >30 mm); stage (stage I-II or Stage III-IV); major post-operative complications (yes or not). Specifically, we sought to match each SVATS patient to CVATS patient who had a propensity score that was identical to 9 digits. If this match could not be found, the algorithm then proceeded sequentially to the next highest digit match on propensity score to make "next best" matches, in a hierarchical sequence until no more matches could be made. Once a match was made, previous matches were not reconsidered before making the next match. Initial SVATS and CVATS group were composed by 84 and 139 patients, respectively. Particularly SVATS group was composed by 51.2% males and 48.8% females, with mean age 63.3 year-old. and standard deviation 1.8 year-old, while CVATS group was composed by 71.2% males and 38.8% females, with mean age 62.9 year-old, and standard deviation 7.2 year-old. After propensity score matching, two new groups were defined both with 61 patients for SVATS and CVATS group. In this case SVATS group was composed by 49.2% males and 50.8% females, with mean age 63.1 year-old. and standard deviation 3.8 year-old, while CVATS group was composed by 50.8% males and 49.2% females, with mean age 63.2 year-old, and standard deviation 8.2 year-old. We observed that first the matching there was a significant difference between propensity score of the groups (0.55±0.20 vs. 0.27±0.23, p-value < 0.0001 with T-test test). Instead after propensity score matching with logit model, we have defined two groups with same numerously, equal to 61. For these groups there was not a significant difference between propensity score (0.48±0.14 vs. 0.48±0.12, p-value = 0.98 with T-test test) as showed in Figure 1

**Figure 1.** Propensity score graph first (SVATS and CVATS group) and after propensity score matching (SVATS_PSM and CVATS_PSM group).
